# Copper–Silver Nanoparticle/Lipase Nanobiohybrids for Enhanced Activity Against Viral Pathogens

**DOI:** 10.1021/acsanm.5c01377

**Published:** 2025-05-13

**Authors:** Clara Ortega-Nieto, Ángela Vázquez-Calvo, Mayte García-Castey, Antonio Alcamí, Jose M. Palomo

**Affiliations:** † 83076 Instituto de Catálisis y Petroleoquímica (ICP), CSIC, C/Marie Curie 2, 28049 Madrid, Spain; ‡ Centro de Biología Molecular Severo Ochoa, Consejo Superior de Investigaciones Científicas (CSIC)-Universidad Autónoma de Madrid (UAM), 28049 Madrid, Spain

**Keywords:** virucidal agent, silver nanoparticles, copper nanoparticles, biomaterial, viruses, nanobiotechnology

## Abstract

The development of sustainable, low-toxicity materials that are effective against a wide range of microorganisms is crucial in addressing emerging infectious diseases. The recent spread of monkeypox virus (MPXV), respiratory pathogens such as rhinoviruses or seasonal coronaviruses, and animal pathogens such as porcine reproductive and respiratory syndrome virus (PRRSV) highlights the urgent need for innovative solutions in both human and animal health. In this study, we designed a bimetallic nanobiohybrid material, NanoCuAg, composed of a lipase and in situ-synthesized copper and silver nanoparticles, with a low silver-to-copper ratio, through a simple and sustainable synthetic process. The nanobiomaterial, featuring a supramolecular flower structure containing ∼4 nm average diameter nanoparticles, contains 32% copper and 3% silver, mainly in the Cu­(II) and Ag­(I) oxidation states. Despite its low silver content, the nanobiomaterial showed a strong catalytic efficacy in different model reactions. Then, its virucidal activity was evaluated under different conditions. At 200 ppm, in combination with hydrogen peroxide, it inactivated 99% of human rhinovirus B14 and 99.99% of human coronavirus 229E. At 1000 ppm, it achieved 90% efficacy against MPXV and a 4.8 log_10_ (≈99.999%) reduction in PRRSV. These results demonstrate the potential of NanoCuAg as a highly effective virucidal material, capable of inactivating both enveloped and nonenveloped viruses at low concentrations, making it a promising candidate for broad-spectrum virucidal applications.

## Graphical abstract

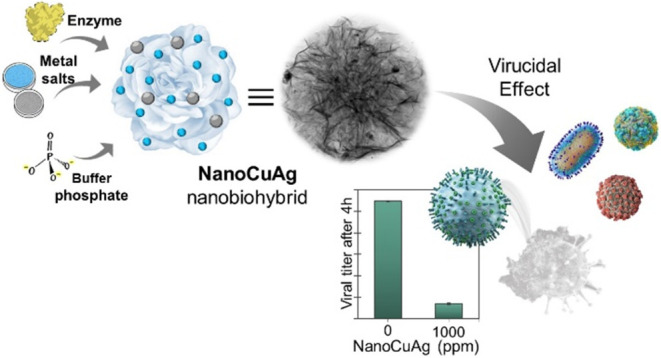

## 1 Introduction

In recent years, the global scientific community has focused on the development and discovery of novel materials with enhanced antimicrobial properties, minimal toxicity, and sustainable compositions. The growing interest in this field is driven by the emergence or the increasing prevalence of infectious diseases, including highly contagious viruses characterized by rapid transmission and significant public health impact.^1^

Monkeypox virus (MPXV) serves as a notable example. In the past few years, cases of monkeypox have been increasingly reported in countries where the disease is not endemic. Although this enveloped virus is primarily transmitted through close contact with an infected person, it can also spread via contaminated objects or within community settings.^2–4^ The World Health Organization (WHO) has recognized the severity of this issue, declaring monkeypox a Public Health Emergency of International Concern twice in the past three years.^5^

Other respiratory pathogens, such as different human rhinoviruses and common seasonal coronaviruses, affect thousands of individuals annually. Although they are usually associated with the common cold, increasing research suggests that they may also be linked to more severe illnesses.^6–8^

However, viruses that infect humans are not the only ones that are drawing the attention of the international community. Viruses that infect animals also pose significant challenges. One example is porcine reproductive and respiratory syndrome (PRRS), which is a significant issue for the pig farming sector. The PRRS virus (PRRSV), an enveloped virus, is endemic in most pig-producing countries and causes substantial economic losses worldwide. Despite its high susceptibility to environmental factors and disinfectants, PRRSV is characterized by a remarkable ability to mutate.^9,10^ New strains emerge annually, affecting farms across extensive regions and compounding the ongoing challenges faced by the pork industry.^11^

Consequently, the development of new mechanisms and materials for the sustainable control of these problematic viruses is crucial from multiple perspectives.

In response, researchers have explored a broad spectrum of strategies, including advanced antimicrobial coatings, nanotechnology-based interventions, and environmentally friendly disinfection methods. These approaches often utilize metals, synthetic polymers, biopolymers, carbon-based compounds, biomolecules, and their combinations due to their recognized virucidal properties.^12–14^

In particular, the use of metal nanoparticles has proven to be very effective in the treatment of viruses due to their special properties and wide range of applications.^15–18^ A number of metals, such as silver and copper, have demonstrated remarkable virucidal efficacy, paving the way for new strategies to combat infections.^19–24^ Indeed, some copper-based products are already on the market for this purpose, although these contain copper in various forms, mainly CuO and bulk copper.^19,20,25^ This raises several questions due to the exceptionally high concentration of copper involved. Moreover, the effectiveness of some copper-based species is dependent on their ability to release copper ions. While copper ions are known to inactivate viruses by targeting their nucleotide sequences, a different mechanism is required when the complete destruction of the viral particle is necessary. Viral inactivation appears to be associated with the generation of reactive oxygen species (ROS) (Figure S1), and the presence of oxidative agents such as peroxide can enhance the biocidal activity of copper. For instance, enveloped viruses require an initial disruption of the lipid membrane, followed by multiple inhibitory actions mediated by copper. These include the inhibition of viral proteases, the inactivation of viral metalloproteins, binding to viral nucleotides, cross-linking of nucleic acid strands, or their fragmentation—processes that are irreversible due to the absence of nucleic acid repair mechanisms.^26^

Furthermore, the utilization of combinations of different metals during the synthesis of nanoparticles provides numerous benefits in comparison to nanoparticles composed of a single metal.^19,27^ These benefits encompass the enhancement of the properties.

Most commercial materials contain copper or silver, especially oxides. But this approach is inefficient and costly. So, new nanostructured materials combining both metals are being developed. The incorporation of silver as a secondary metal is a suitable solution for this purpose due to its virucidal properties. The combination of two metals has been shown to produce synergistic effects, thereby enhancing the activity of bionanohybrids.^17^ However, it should be noted that a majority of applications necessitate the use of substantial quantities of silver in the form of nanoparticles.

Their instability causes unfavorable agglomeration and/or decomposition. A challenge is developing bimetallic systems with a minimal silver content while maintaining efficiency. This requires controlling and reducing particle sizes, ensuring optimal dispersion, high stability, and preventing aggregation. This method maximizes the exposure of active sites on the nanoparticle surface, thereby improving the performance. Within this framework, employing a biologically derived molecule as part of an enzyme-mediated approach to synthesize metal nanoparticles within a protein matrix under mild conditions represents an optimal strategy.^28–31^ This method offers distinct advantages—particularly in generating highly catalytic bimetallic systems—when compared to alternative approaches that involve synthesizing bimetallic nanoparticles in solution or on various materials.^17,32^

A key feature of this eco-friendly technology is the use of the enzyme as a stabilizer, enabling the nanoparticles to form directly under gentle synthesis conditions. This process often eliminates the need for a reducing agent, making it more environmentally friendly. The outcome is the generation of highly efficient materials developed under sustainable conditions by using sustainable components.

Copper flower-like structures are highly effective against some viruses.^33,34^ A novel system utilizing Cu-flower as a supramolecular scaffold allowing for the effective formation of ultrasmall AgNPs with a high concentration of active sites on its surface could offer a superior alternative.

A novel approach to creating a sustainable and stable bimetallic nanoparticle system is proposed, with the focus of this study being the formation of stable Ag nanoparticles on the surface of a Cu phosphate-nanoflower macrostructure (Figure 1a).

This study describes the design and synthesis of a bimetallic copper–silver nanobiohybrid utilizing a highly stable commercial lipase as a structural scaffold. Its virucidal properties were evaluated against viruses of concern to health authorities. Notably, a small proportion of silver is used relative to copper (1:18 weight ratio), aiming to achieve a synergistic virucidal effect while minimizing the amount of silver used.

## 2 Experimental Section

### 2.1 Chemicals

Lipase B from Candida antarctica (CALB) solution (Lipozyme CalB) was obtained from Novozymes (Copenhagen, Denmark). Copper­(II) sulfate pentahydrate [Cu_2_SO_4_·5H_2_O], hydrogen peroxide (H_2_O_2_, 33%), and sodium hydroxide (NaOH) were obtained from Panreac (Barcelona, Spain). Sodium dihydrogen phosphate dihydrate (NaH_2_PO_4_·2H_2_O), *p*-nitrophenol, *p-*aminophenol, l-ascorbic acid, l-histidine, and sodium borohydride (NaBH_4_) were obtained from Sigma-Aldrich (St. Louis, MO). Silver nitrate (AgNO_3_) was provided by Thermo Fisher Scientific (Waltham, MA).

### 2.2 Cells and Viruses

HuH-7 cells (a generous gift from Isabel Solá and Luis Enjuanes, CNB-CSIC, Madrid, Spain), HeLa-H1 cells (kindly provided by Mauricio Garca-Mateu, CBMSO, Madrid, Spain), MARC-145 cells (courtesy of Cinta Prieto, Universidad Complutense de Madrid, Spain), and BSC-1 cells (obtained from the Collection [ATCC], CCL-26 American Type Culture) were cultured in Dulbecco’s Modified Eagle’s Medium (DMEM; Invitrogen) supplemented with 5% fetal bovine serum (FBS), 2 mM l-glutamine, 100 mg/mL streptomycin, and 100 U/mL penicillin and maintained at 37 °C with 5% CO_2_. The viruses used in this study included HCoV-229E (generously provided by Isabel Solá and Luis Enjuanes, CNB-CSIC, Madrid, Spain), HRV14 (courtesy of Mauricio García-Mateu, CBMSO, Madrid, Spain), PRRSV (provided by Cinta Prieto), and MPXV. All procedures involving infectious viruses were conducted in biosafety level 2 (BSL-2) or BSL-3 laboratories.

### 2.3 General Synthesis of NanoCuAg Hybrid

3.6 mL portion of CALB solution (from a 10 mg/mL commercial solution) was added to 60 mL of a sodium phosphate 0.1 M pH 7 solution in a glass bottle containing a small magnetic bar stirrer. Then, 540 mg of Cu_2_SO_4_·5H_2_O (10 mg/mL) and a total of 30 mg of AgNO_3_ was introduced into the protein solution, which was then incubated for 24 h. In the first hour, a dark bluish solution was produced, which turned dark gray after 24 h. Then, the mixture was centrifuged for 8 min at 6440*g*. After the supernatant was discarded, the pellet was rinsed with distilled water, a step that was repeated three times. The resulting solid was then resuspended in 2 mL of distilled water in a cryotube, flash-frozen using liquid nitrogen, and lyophilized for 16 h.

### 2.4 Characterization and Analysis Methods

X-ray diffraction (XRD) patterns were obtained using a PANalytical X’Pert Pro polycrystalline X-ray diffractometer with a D8 Advance texture analysis θ–θ setup (Malvern Panalytical Ltd., Malvern, U.K.) with radiation Cu Kα (λ = 1.5406 Å, 45 kV, 40 mA). Their analysis was performed using the X’Pert Data Viewer and X’Pert Highscore Plus programs. Transmission electron microscopy (TEM) was conducted using an S/TEM Titan 80-300 microscope equipped with a Cs probe corrector and energy-dispersive spectroscopy (EDS) for elemental analysis. Samples were prepared by dispersing the material in ethanol and placing a drop on a carbon-coated nickel grid, followed by drying and plasma cleaning. Imaging was performed in both TEM (BF, DF, and SAED) and STEM modes (BF and HAADF detectors). Scanning electron microscopy (SEM) was carried out using a Hitachi TM-1000 microscope with samples mounted on conductive carbon tape. X-ray photoelectron spectroscopy (XPS) was performed using a SPECS system under ultrahigh vacuum equipped with a PHOIBOS 150 9MCD analyzer and monochromatic X-ray source.

Their analysis was carried out using CasaXPS program. Inductively coupled plasma-optical emission spectrometry (ICP-OES) was performed by using an AVIO 220MAX instrument (PerkinElmer, Waltham, MA). Five milligrams of the solid sample was treated with 6 mL of HCl (37% v/v) for acid leaching. Then, 44 mL of water was added, the sample was centrifuged, and the clear solution was analyzed for Cu and Ag content. ICP-OES analyses were performed in duplicate with three replicates of each sample. Fourier transform infrared spectroscopy (FT-IR) was performed using an FT-IR-4600 spectrophotometer (JASCO, Tokyo, Japan). Chromatographic analyses were performed on an HPLC system equipped with a pump (PU-4180, JASCO, Tokyo, Japan) and a UV-4075 ultraviolet–visible spectroscopy (UV–Vis) detector (JASCO, Tokyo, Japan) in an isocratic mode at ambient temperature. Spectrophotometric analyses were run on a V-730 spectrophotometer (JASCO, Tokyo, Japan).

### 2.5 Fenton-like Activity of NanoCuAg

Ten mL portion of a 10 mM *p*-aminophenol (pAP) solution was prepared in distilled water containing H_2_O_2_ (0.5%, v/v). Three milligram of NanoCuAg was used to catalyze the reaction. The mixture was stirred at room temperature under magnetic agitation. Reaction progress was monitored by HPLC analysis of samples collected at different times. Samples were centrifuged and diluted 1:10 with the mobile phase prior to injection. HPLC analysis was carried out using a Gemini-NX C18 column (5 μm, 110 Å, 250 mm × 4.6 mm), a mobile phase of ultrapure water and acetonitrile (90:10) at pH 4, an injection volume of 10 μL, a flow rate of 0.7 mL/min, and UV detection at 210 nm. Under these conditions, the retention times of pAP and H_2_O_2_ were 3.0 and 3.8 min, respectively.

### 2.6 Reductase-like Activity of NanoCuAg

Two milliliters of a 50 mM p-nitrophenol (pNP) solution was prepared in distilled water. 150 mg of NaBH_4_ was added to the solution, which was stirred at room temperature for 30 s to generate the phenolate ion. Then, catalyst (1 mg) was added to the mixture, which was kept under magnetic stirring. Samples were taken at different times, centrifuged, and diluted 1:100 with water in plastic cuvettes (1 cm path length). The reaction was followed by measuring the transformation of pNP to pAP in the range of 290 to 600 nm.

### 2.7 Virus Titrations

Virus yield was determined by plaque assay in HuH-7 cells (HCoV-229E), HeLa-H1 (HRV14), BSC-1 (MPXV), or MARC-145 (PRRSV). Cells were seeded into 12-well plates and incubated at 37 °C for 24 or 48 h (in the case of HuH-7) in 5% CO_2_. Then, the cells were inoculated with 0.2 mL of the serial 10-fold diluted harvested supernatant. The virus inoculum was removed after 1 h of adsorption at 37 °C or 2 h for HCoV-229E. Then, medium containing 0.7% agarose, DEAE-dextran (0.090 mg/mL), and 2% FBS was added, and plates were incubated for 4 days at 33 °C in the case of HCoV-229E. For HRV14 and PRRSV, after inoculum removal, medium containing 0.7% agarose, DEAE-dextran (0.045 mg/mL), and 2% FBS was added, and plates were incubated for 3 days at 35 °C (HRV14) or 37 °C (PRRSV). In the case of MPXV, after inoculum removal, infections were allowed to proceed in semisolid medium containing 1.5% carboxymethylcellulose, 10 mM HEPES, and 2% FCS, and plates were incubated at 37 °C for 4 days. At this point, cells were fixed in 4 or 10% formaldehyde for at least 30 min at room temperature and stained with 3% crystal violet in 2% formaldehyde. Finally, the plates were washed, and the viral plates were counted.

### 2.8 Virucidal Assay

To determine the virucidal activity on different viruses, the nanobiomaterial was incubated directly with the viruses. Brieftly, 100 μL of PBS containing ∼10^5^ plaque forming units (PFU) of HCoV-229E, HRV14, PRRSV, or MPXV were incubated with different concentrations of NanoCuAg (0, 200, or 1000 ppm) without or with 0.5% H_2_O_2_ in a rotatory movement at RT for 4 h in a final volume of 500 μL in PBS. To separate the NanoCuAg particles, the suspensions were centrifuged at 5340*g* at 4 °C for 10 min, and the supernatant was recovered carefully and stored at −80 °C until viral titration. Finally, the virus viability after treatment was determined by virus titration, as described previously.

**Figure 1:**
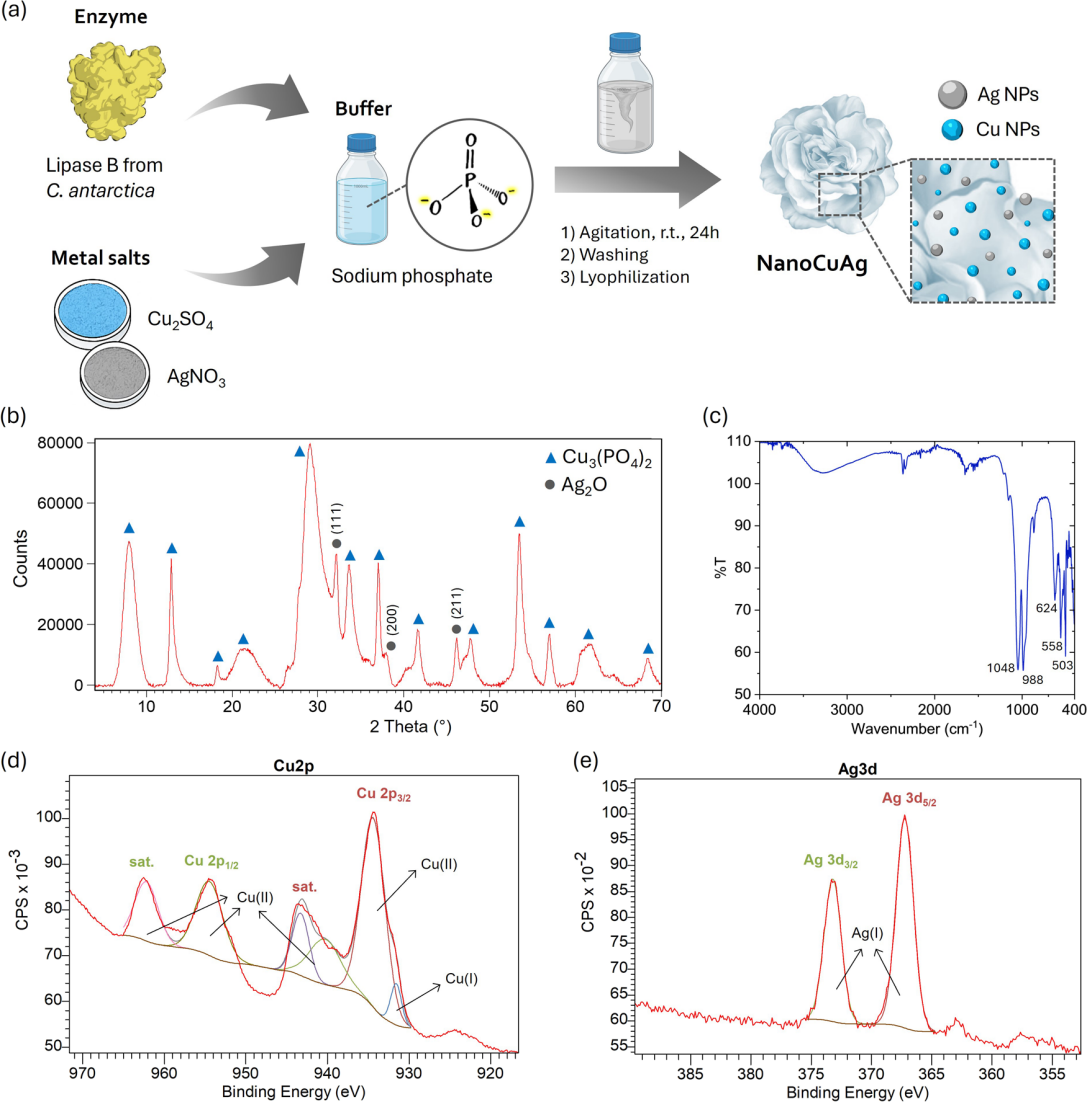
Synthesis and characterization of NanoCuAg. (a) Scheme of synthesis. (b) X-ray diffraction pattern. (c) FT-IR spectrum. (d, e) XPS high-resolution spectra of Cu 2p and Ag 3d, respectively.

## 3 Results and Discussion

### 3.1 Synthesis and Characterization of NanoCuAg

The NanoCuAg hybrid was prepared at room temperature in an aqueous solution, following the method outlined in Section 2. In summary, a CALB solution was mixed with copper sulfate and silver nitrate in phosphate buffer to generate the bimetallic nanomaterial (Figure 1a), which was fully characterized. First, ICP-OES analyses were carried out to determine the metal content of the new nanobiohybrid, which revealed a copper content of 32.0 ± 0.9% (w/w) and a silver content of 2.7 ± 0.1% (w/w). Then, the metallic species present in the hybrid were studied by XRD. In the obtained pattern (Figure 1b), peaks matching the copper phosphate standard (JCPDS card no. 00-022-0548) were identified.

In addition, it was determined that the peaks at 32, 38, and 46° exhibited a strong correlation with those reported for Ag_2_O (JCPDS no. 00-076-1393), in particular with (111), (200), and (211) indices, respectively. This observation is consistent with the findings reported in the biological synthesis of Ag_2_O NPs.^35,36^ FT-IR analysis showed two bands in the P–O stretching region, at 1048 and 988 cm^–1^, which corroborates the presence of phosphate in the hybrid (Figure 1c). Moreover, the bands present in the range 400–650 cm^–1^ are attributable to Me–O vibrations.

To prove that copper phosphate and silver oxide were the only species present in the nanobiomaterial, X-ray photoelectron spectroscopy (XPS) was conducted. The full survey spectrum (Figure S1a in the electronic supporting information (ESM)) confirmed the existence of Cu, Ag, O, and P elements. The high-resolution XPS spectra of Cu 2p (Figure 1d) showed two prominent peaks that appear at 934.4 and 954.5 eV, corresponding to Cu 2p_3/2_ and Cu 2p_1/2_. The Cu 2p_3/2_ peak can be deconvoluted into two components at 934.4 and 931.6 eV, attributed to Cu­(II) and Cu­(I) states, respectively.^37,38^ Additionally, three “shake-up” satellite peaks are observed at 962.2, 943.3, and 940.4 eV, which indicate the presence of Cu­(II), as many authors report.^37,39^

The main and satellite peaks indicate that Cu­(II) is the predominant species, comprising over 99% of the total copper content. The minor contribution from Cu­(I), less than 1%, could indicate a slight oxidation of Cu on the surface of the nanobiohybrid. The O 1s spectrum shows a unique peak at 530.4 eV (Figure S1b), which is consistent with the reported values for Ag_2_O^40^ in the literature.

The detailed XPS spectra of Ag 3d (Figure 1-e-) exhibited two binding peaks at 373.2 and 367.2 eV, which correspond to Ag 3d_3/2_ and Ag 3d_5/2_, respectively. These values are in good agreement with those reported in the literature for Ag_2_O.^40,41^ SEM images revealed that NanoCuAg possessed a microflower-like shape of 25 ± 13 μm (Figures 2a,b and S2). A more in-depth study of the morphology was conducted using TEM (Figures 2c–e and S3), which revealed that the microflower morphology persisted even at nanometric sizes. The selected area electron diffraction (SAED) pattern of the slice shown in Figure S3d reveals that the crystalline phase of the hybrid material is polycrystalline, as indicated by the simultaneous presence of diffraction rings and discrete spots. In addition, calculations from SAED analysis are in good agreement with those from the JCPDS card for Ag_2_O (Figure S3e). Additionally, the formation of well-dispersed metal NPs with an average size of 4.1 ± 0.2 nm was observed. High-angle annular dark-field scanning transmission electron microscopy (HAADF-STEM) showed that these small particles corresponded mainly to Cu NPs, with a minor presence of Ag (Figure S4a). This observation agrees with the abundance data obtained by ICP-OES. Furthermore, some particles larger than 10 nm, around 50–60 nm, were observed, which, according to HAADF-STEM analysis, would correspond mainly to Ag (Figure S4c).

Previous experiments showed that the addition of silver to an earlier formed copper nanoflower produced Ag­(I) NPs in the form of silver phosphate, with an exchange between Cu and Ag occurring upon the addition of the silver salt.^42^ However, this new synthesis method produces Ag_2_O NPs. Therefore, it is hypothesized that a Cu microflower is rapidly formed together with the phosphate and the enzyme as the scaffold, with Ag coordinating in second place. In this case, the protein has no reducing capacity, since it is involved in the formation of the microflower, but no Cu–Ag exchange takes place, so the silver is oxidized, and Ag_2_O NPs are formed.

**Figure 2:**
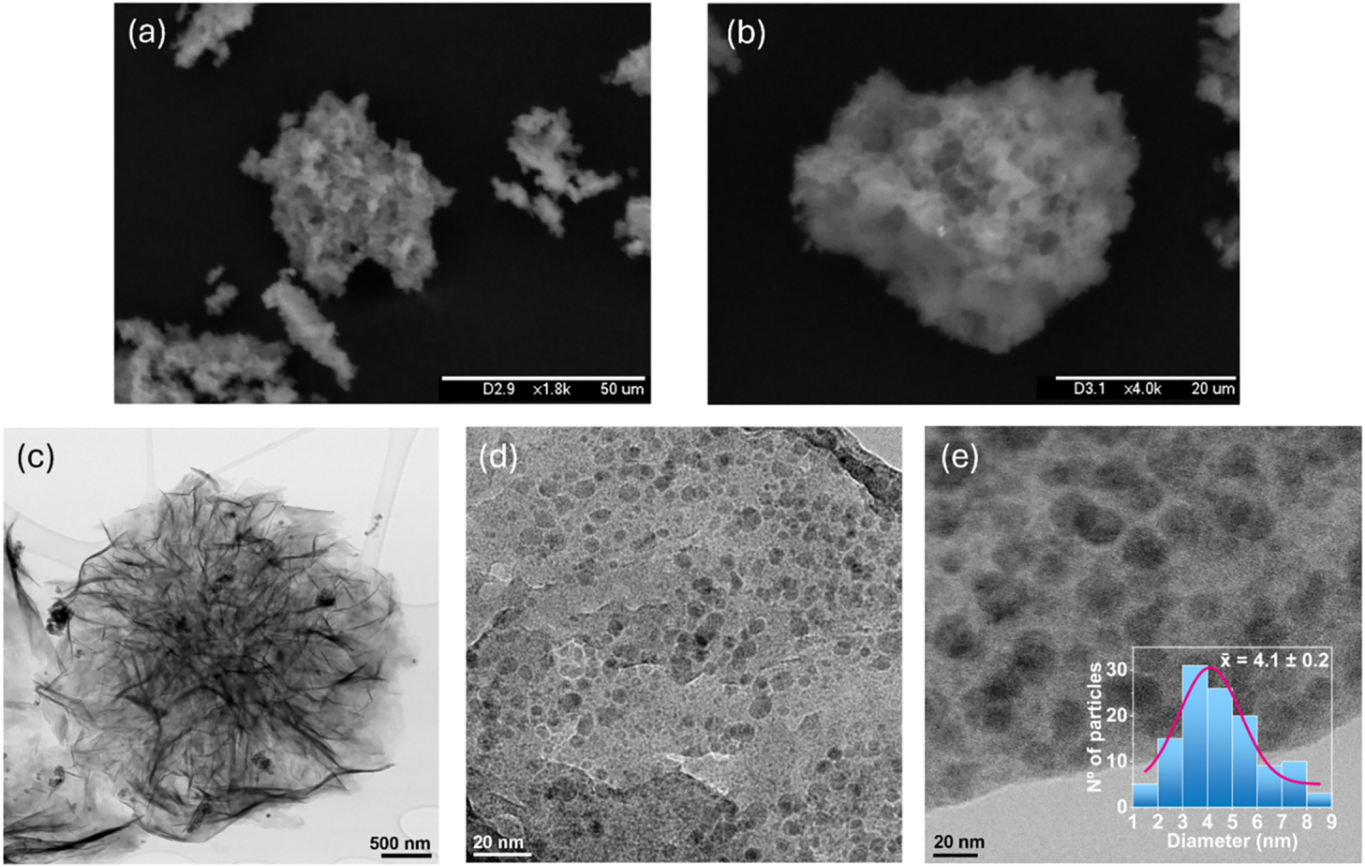
Microscopic characterization of NanoCuAg. (a, b) SEM images showing the surface morphology. (c–e) TEM images providing detailed structural information (inset in e: particle size distribution fitted by a Gaussian curve).

### 3.2 Evaluation of Fenton-like Activity and Reductase-like Activity of NanoCuAg

The metallic activity of NanoCuAg was evaluated by using two different model reactions to assess the formation of metallic particles. Initially, the fenton-like catalytic activity was evaluated by monitoring the selective hydroxylation of *p*-aminophenol to benzoquinone, employing hydrogen peroxide as an environmentally friendly oxidizing agent.

This reaction serves as an indirect mimetic assay of the reactive oxygen species (ROS) capacity of the hybrid, a method recently employed to confirm the antibacterial properties of various MeNPs–enzyme hybrids.^30,42^ As shown in Figure 3(a), the reaction achieved almost 80% conversion within 5 min, reaching 100% conversion after 10 min.

Second, its reductase-like activity was examined via the transformation of *p*-nitrophenol (pNP) to pAP (Figure 3(b)). Since Cu­(II) is inactive in this reaction,^43^ this activity specifically confirms the catalytic role of silver. Results indicated that the reaction reached 50% of conversion in 1 min and achieved 95% conversion in 5 min.

In terms of turnover frequency (TOF), considering the silver content of the nanobiomaterial as the active site, a TOF of 3.5 s^–1^ was calculated over a 5 min period. This indicates that each active site catalyzed 3.5 reactions per second throughout the reaction duration. This is an indication of the high efficiency of silver despite its low content in the hybrid.

**Figure 3:**
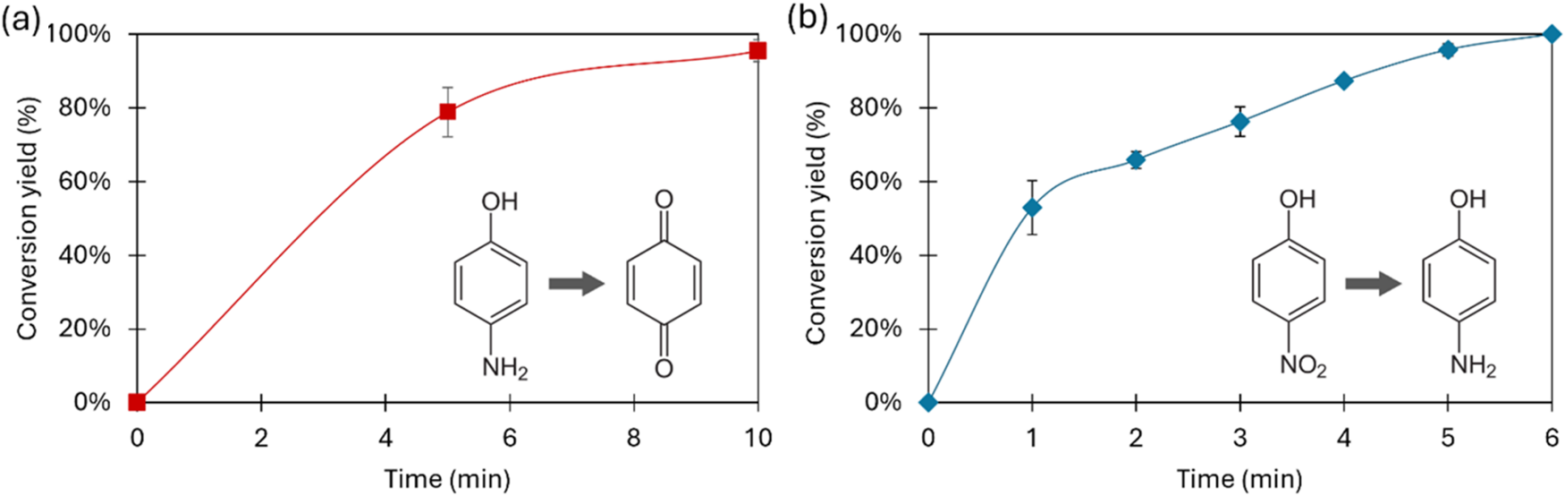
Reaction profiles of model reactions catalyzed by NanoCuAg. (a) Fenton-like activity. The oxidation of 10 mM pAP was catalyzed by 3 mg of NanoCuAg in the presence of 0.5% H_2_O_2_ (v/v). Data represent the mean ± SD of four experiments. (b) Reductase-like activity. The reduction of pNP 50 mM was catalyzed by 1 mg of NanoCuAg in the presence of NaBH_4_. Data represent the mean ± SD of three experiments.

### 3.3 Virucidal Properties of NanoCuAg

To assess the potential of NanoCuAg to reduce viral infectivity, a suspension of the hybrid material was incubated with different viruses under rotary shaking at room temperature. Virucidal activity was determined using plaque assays, a standard method for evaluating viral titration of infectious particles (PFU).

First, the virucidal activity was tested against human coronavirus 229E (HCoV-229E) and human rhinovirus B14 (HRV14). Different concentrations of NanoCuAg (200 and 1000 μg/mL), as well as its use in the presence of 0.5% hydrogen peroxide, were evaluated, following a 4 h exposure period (Figure 4).

**Figure 4:**
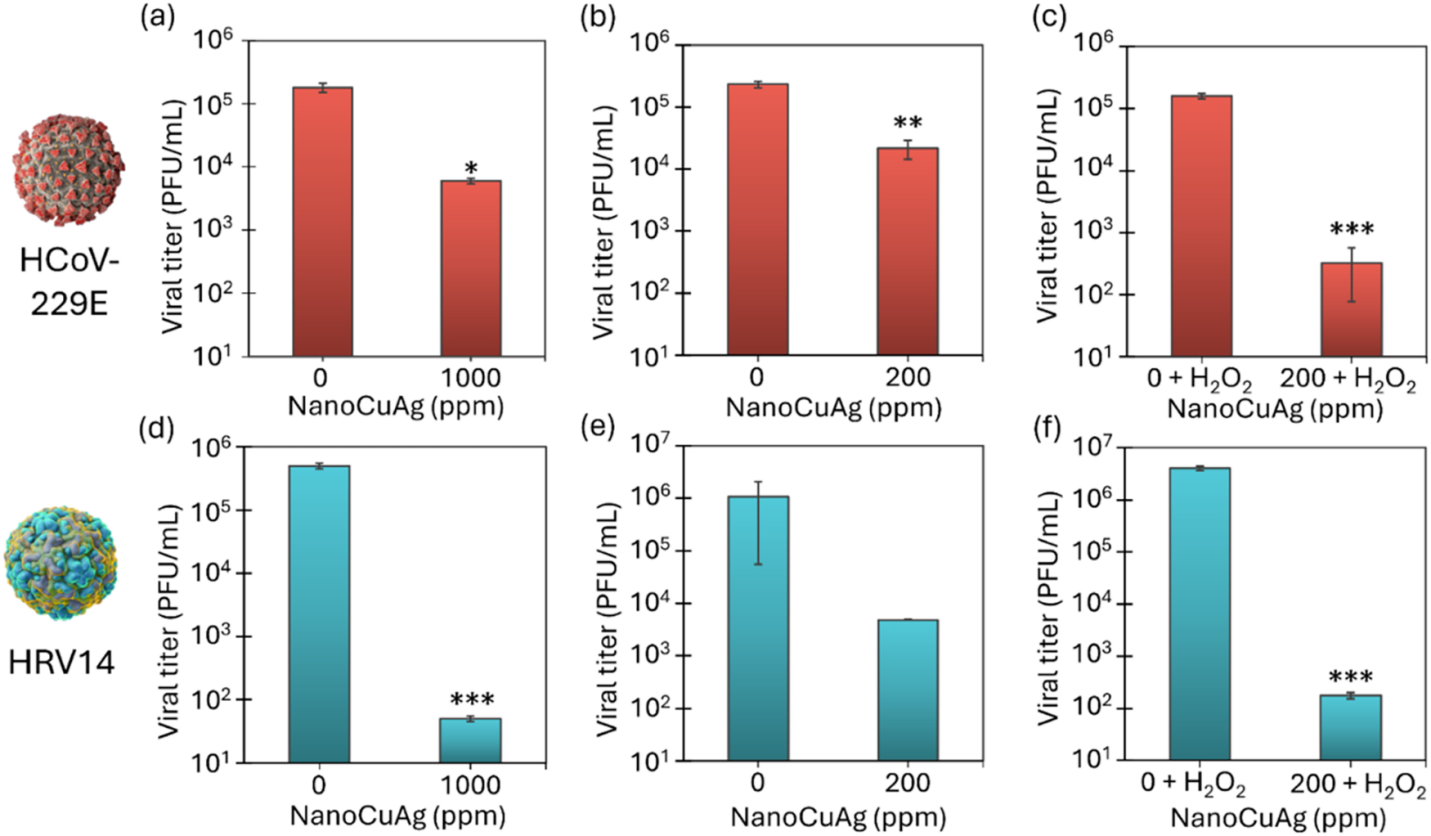
Virucidal Effect of NanoCuAg against different viruses. Approximately 10^5^ PFU of HCoV-229E (a–c) or 10^6^ PFU of HRV14 (d–f) were incubated with different concentrations of NanoCuAg with or without 0.5% (v/v) H_2_O_2_ for 4 h: (a, d) 1000 μg/mL of NanoCuAg; (b, e) 200 μg/mL of NanoCuAg; (c, f) 200 μg/mL of NanoCuAg + H_2_O_2_. Controls containing 0.5% (v/v) H_2_O_2_ were made in parts (c) and (f). Means ± SD from two independent assays are represented. Unpaired *t* test analysis was employed for comparing experimental treatments with the controls. The black asterisks indicate significant differences between the control and the respective treatment (*p < 0.05, **p < 0.01, ***p < 0.005).

Results demonstrated that at a concentration of 1000 ppm, the hybrid reduced HCoV-229E by 1.5 log_10_ (Figure 4(a)). When the concentration was reduced to 200 ppm, the log_10_ reduction was 1.0, as represented in Figure 4(b). This indicates that a fivefold reduction in the concentration only reduces the virucidal effect 1.4-fold against the tested coronavirus. In contrast, NanoCuAg showed significantly stronger virucidal activity against the nonenveloped HRV14.

At 1000 ppm, NanoCuAg reduced the virucidal titer of HRV14 by 4.0 log_10_ (Figure 4d). Even at the lower concentration of 200 ppm, a reduction of 2.7 log_10_ was achieved (Figure 4e). These results indicate the good antimicrobial activity of nanohybrid even at very low concentrations, such as 200 ppm.

In order to improve the efficiency of the nanomaterial, its virucidal effect at 200 ppm was evaluated with the incorporation of 0.5% H_2_O_2_ (v/v) as an additive (Figure 4c,4f) against both viruses after 4 h of exposure. In the case of HCoV-229E, the combination of the two elements produced a viral reduction of 2.3 log_10_, which was more effective than using a concentration of 1000 ppm of NanoCuAg alone. For HRV14, the virucidal effect of the hybrid at 200 ppm was significantly enhanced in the presence of H_2_O_2_, achieving a logarithmic reduction of 4.4, corresponding to a 99.99% decrease in the vial load. This reduction was also greater than the effect observed against HRV14 with a concentration of 1000 ppm of the hybrid alone.

Overall, the addition of a small percentage of hydrogen peroxide significantly enhanced the virucidal effect of the copper–silver hybrid, whereas the presence of this oxidant in self-mass did not affect the viruses (Figure 4c,f). This could be explained by considering the fenton-like activity of the catalyst, which generates superoxide anions, hydrogen peroxide (H_2_O_2_), and hydroxyl radicals (^•^OH). The direct presence of hydrogen peroxide increases the number of oxidative species in the mixture, allowing the reaction to first target the capsid proteins (in enveloped viruses), followed by the RNA sequences and functional proteins of the viruses.

In order to validate the ROS formation, the Fenton reaction of pAP (Figure 3) was used as a model in the presence of different scavengers. Sodium azide, which is able to catch hydroxyl radical and superoxide groups, and l-histidine, specific for the superoxide radical (Figure S5). The results showed a decrease of more than 50% in the efficiency of the hybrid in the presence of histidine and about 25% in the presence of sodium azide (Figure S5), suggesting that both oxidative species are generated in the solution due to the activity of the CuAg hybrid.

Finally, the virucidal efficacy of NanoCuAg was assessed against two viruses that are of significant concern to health authorities: MPXV and PRRSV. These viruses were selected due to their global impact on human and animal health. For this evaluation, the nanobiohybrid was tested at a concentration of 1000 ppm, with a 4 h incubation period in direct contact with the viral suspensions. The results showed that NanoCuAg exhibited an effective virucidal activity against MPXV, achieving a viral reduction of over 90%, which corresponds to a log_10_ reduction of 1.3 (Figure 5a).

**Figure 5:**
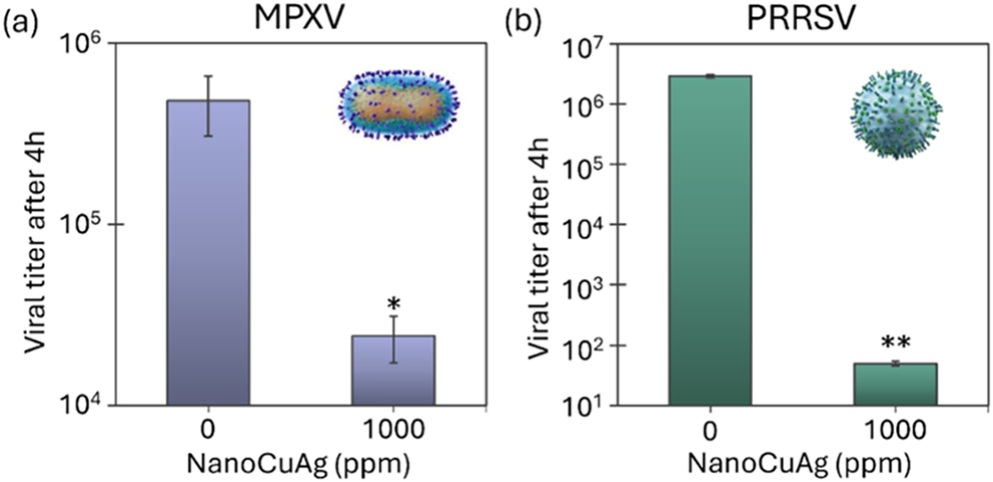
Virucidal effect of NanoCuAg against (a) monkeypox virus (MPXV) and (b) porcine reproductive and respiratory syndrome virus (PRRSV). Approximately 10^5^ PFU of MPXV or 10^6^ PFU of PRRSV were incubated with a 1000 ppm concentration of NanoCuAg for 4 h. Means and SD from the two technical replicates are represented. Unpaired *t* test analysis was employed for comparing experimental treatments with the controls. The presence of black asterisks denotes substantial disparities between the control group and the designated treatment group (*p < 0.05, **p < 0.001).

This outcome is attributed to the unique structural complexity of the Mpox virus, which features a dumbbell-shaped nucleocapsid enclosed within ovoid, lipid-containing particles. Notably, poxvirus virions differ from other enveloped viruses due to their relatively low lipid content in the envelope, making them less susceptible to organic solvents and disinfectants.^44^ In contrast, the virucidal efficacy of the hybrid was remarkably higher against PRRSV. The treatment resulted in a log_10_ reduction of 4.8, equivalent to a viral load reduction of almost 99.999% (Figure 5b).

Additionally, the stability of this hybrid was evaluated, showing very high store stability, conserving 86% of efficiency after 6 months at room temperature on the bench and also in thermal conditions, with more than 99% efficiency after 3 days of incubation at 40 °C (Figure S6).

## 4 Conclusions

Here, we have synthesized and characterized a novel bimetallic nanobiomaterial with exceptional virucidal properties. The hybrid, NanoCuAg, composed of copper and silver NPs generated in situ in a protein matrix, was synthesized using a low silver proportion. Despite the small amount of silver present in the nanomaterial (2.7%, w/w), it exhibited high catalytic performance.

Virucidal tests showed that NanoCuAg was effective against various viruses, including human rhinovirus B14 (HRV14) and human coronavirus 229E (HCoV-229E), even at a low concentration. The virucidal activity was particularly enhanced in the presence of H_2_O_2_, where over 99.99% viral inactivation was achieved against HRV14. This demonstrates the efficacy of the nanobiomaterial against both enveloped and nonenveloped viruses.

Furthermore, NanoCuAg demonstrated its efficacy against two viruses of significant concern to human and animal health: the monkeypox virus (MPXV) and PRRSV virus. For MPXV, the treatment with the hybrid resulted in a 90% of virucidal efficacy, while for PRRSV, a log_10_ reduction of 4.8 was obtained, which translates into almost a 99.999% decrease in the viral load.

The simple and sustainable synthesis process makes NanoCuAg an ideal candidate for industrial applications. Due to its versatile catalytic mechanism, this nanobiomaterial has the potential to inhibit a wide range of viruses, including emerging respiratory pathogens. Taken together, these findings highlight the relevance of this technology in mitigating future viral pandemics as well as addressing global health challenges associated with infectious diseases.

Therefore, one of the potential effective actions may be in the control of virus transmission. This catalytic hybrid material could be an excellent candidate for coating application materials, for example, in fabrics or paints with multiple applications. For instance, the fabrication of medical textiles for utilization in hospital environments, as well as those intended for application in public transportation systems. Forthcoming studies on the scalability of the material, the virucidal capacity on coated surfaces, and their stability in terms of minimum leaching from the support should be considered as the first steps toward future commercialization.

Furthermore, the findings on this research open the door to evaluating this type of bimetallic nanobiohybrid in applications, such as biocorrosion mitigation, wood protection, or water treatment.

## Supplementary Material



## Notes

Experimental results and data are as follows: XPS analysis; additional characterization (SEM and TEM images); HAADF-STEM imaging and STEM-EDX analysis of NanoCuAg; and fenton-like activity of NanoCuAg in the presence of different scavengers and stability graphs (PDF)
