# Supplementary material for: Copper–Silver Nanoparticle/Lipase Nanobiohybrids for Enhanced Activity Against Viral Pathogens
Source: ACS Appl Nano Mater. 2025 May 13;8(20):10559–67. doi: 10.1021/acsanm.5c01377 (PMC12107529; doi:10.1021/acsanm.5c01377)
Supplement: Supplementary file 1 [file an5c01377_si_001.pdf]

Supporting Information

Copper-Silver Nanoparticle/Lipase Nanobiohybrids for Enhanced Activity Against Viral Pathogens

Clara Ortega-Nieto[1], Ángela Vázquez-Calvo[2], Mayte García-Castey[2], Antonio Alcamí[2] and Jose M. Palomo[1*]

[1] Instituto de Catálisis y Petroleoquímica (ICP), CSIC, C/ Marie Curie 2. 28049 Madrid, Spain.
[2] Centro de Biología Molecular Severo Ochoa, Consejo Superior de Investigaciones Científicas (CSIC)-Universidad Autónoma de Madrid (UAM), 28049, Madrid, Spain

*Corresponding author: Jose M. Palomo, josempalomo@icp.csic.es

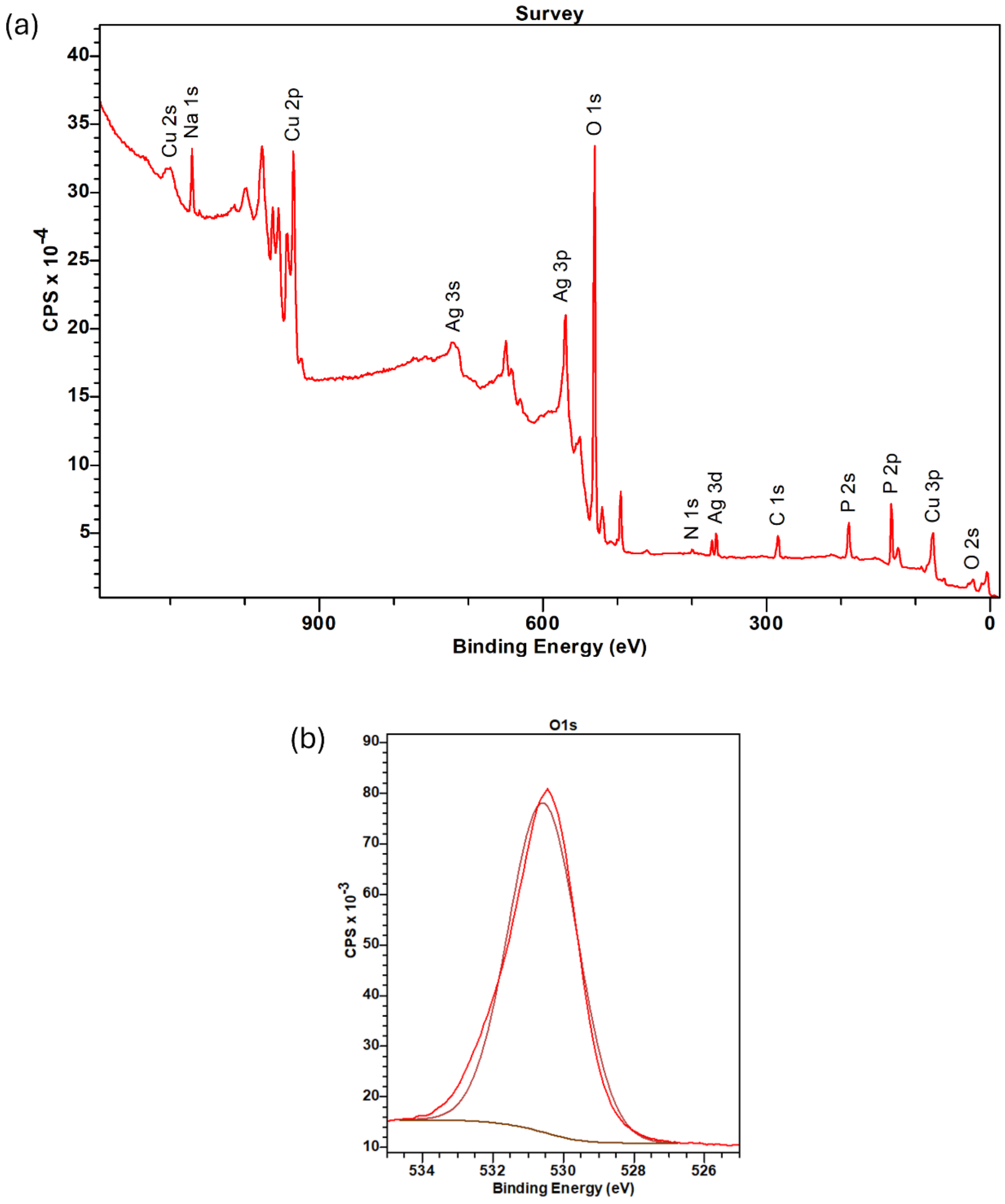

Figure S1. XPS spectra of NanoCuAg (a) Survey. (b) O 1s.

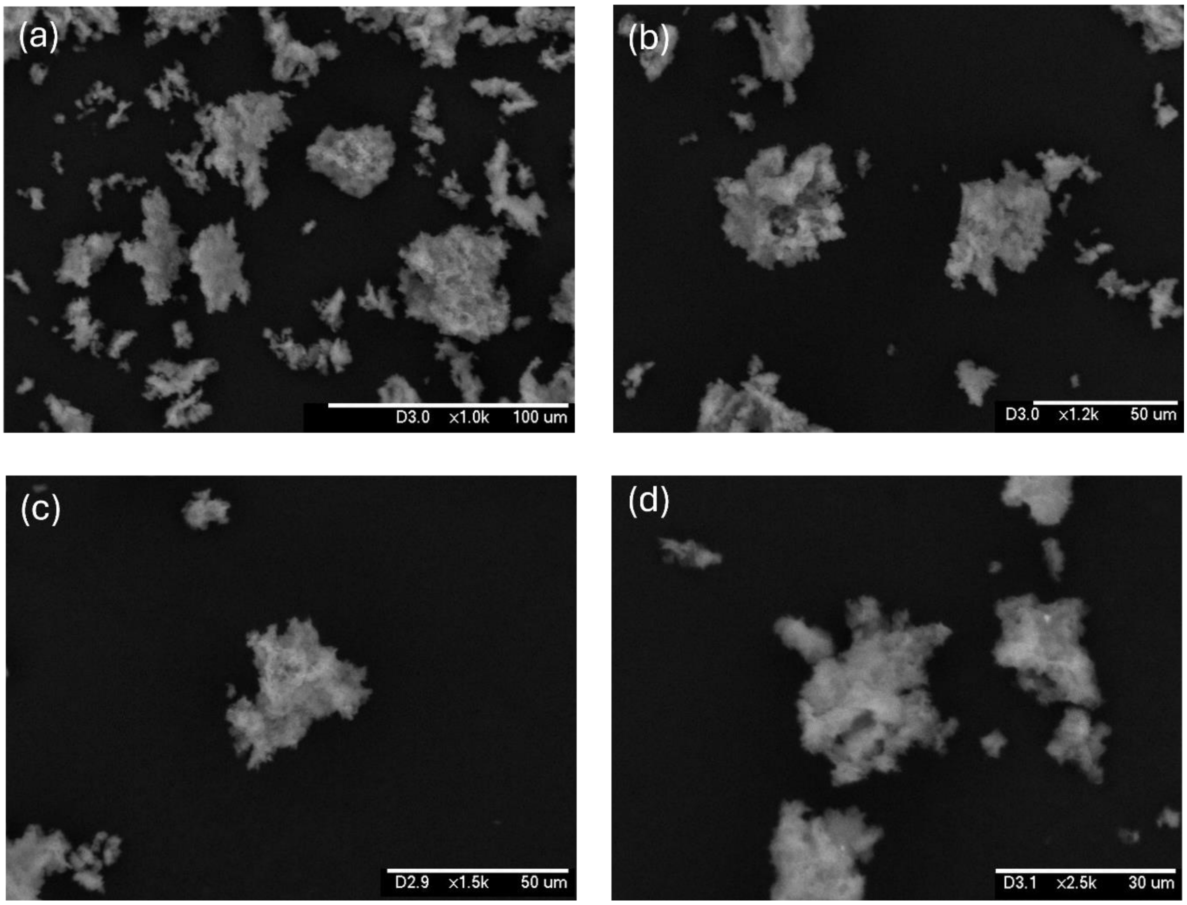

Figure S2. SEM images of NanoCuAg.

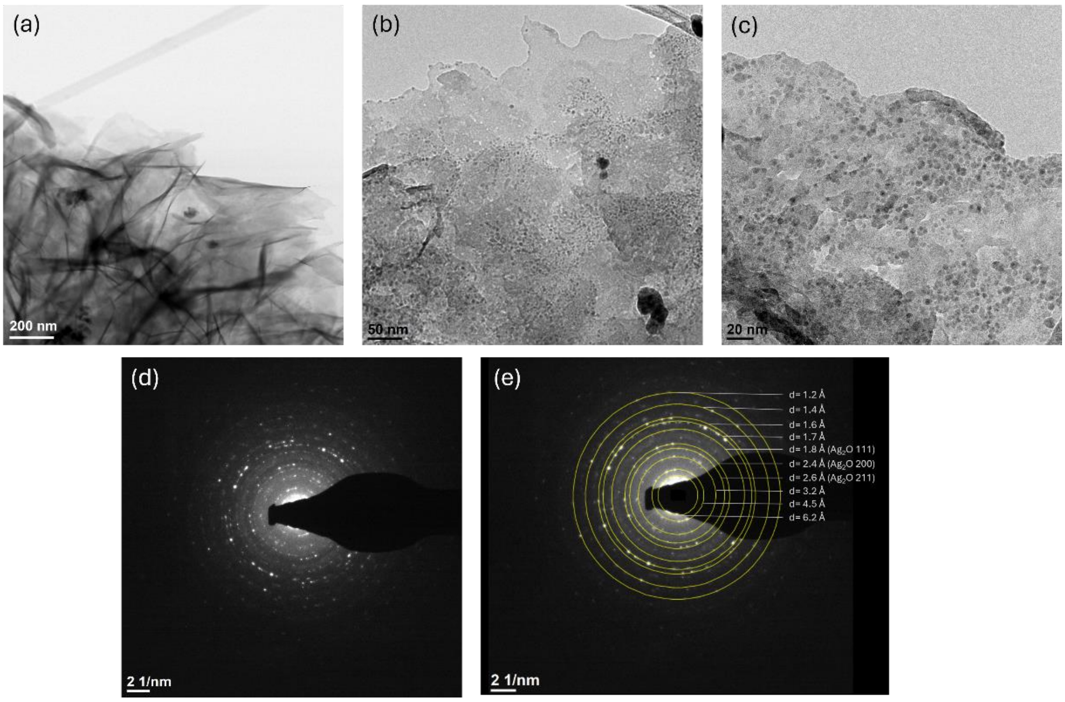

Figure S3. Characterization of NanoCuAg hybrid. (a), (b) and (c) TEM images. (d) Selected area electron diffraction (SAED) pattern. (e) Interplanar spacing and Miller indices from the JCPDS card no. 00-076-1393 calculated from SAED pattern.

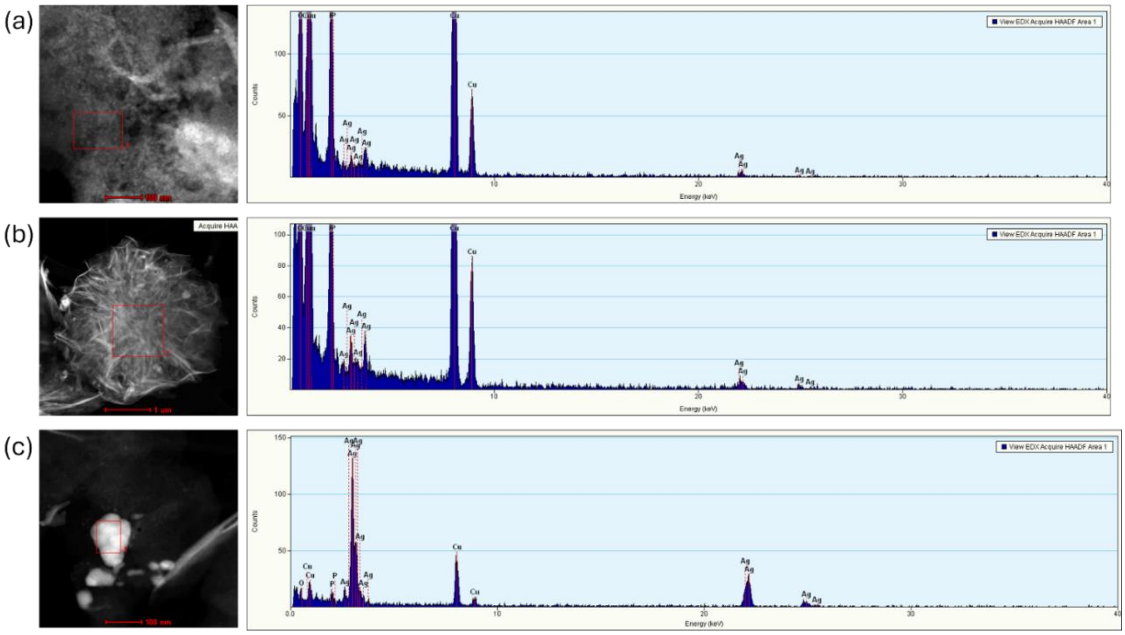

Figure S4. HAADF-STEM imaging (left panels) and STEM-EDX analysis (right panels) of Cu and Ag nanoparticles in NanoCuAg hybrid.

Scavenger agent	[Agent] mM	NanocuAg relative activity (%)
L-histidine	5	46.5
Sodium azide	5	74.4

Figure S5. Effect of the presence of ROS scavengers in the efficiency of NanoCuAg in the oxidation of pAP. The reaction was catalyzed by 3 mg of NanoCuAg in the presence of 0.5% H_2O_2 (v/v). Reaction without scavenger is considered as 100% relative activity after 5 min reaction.

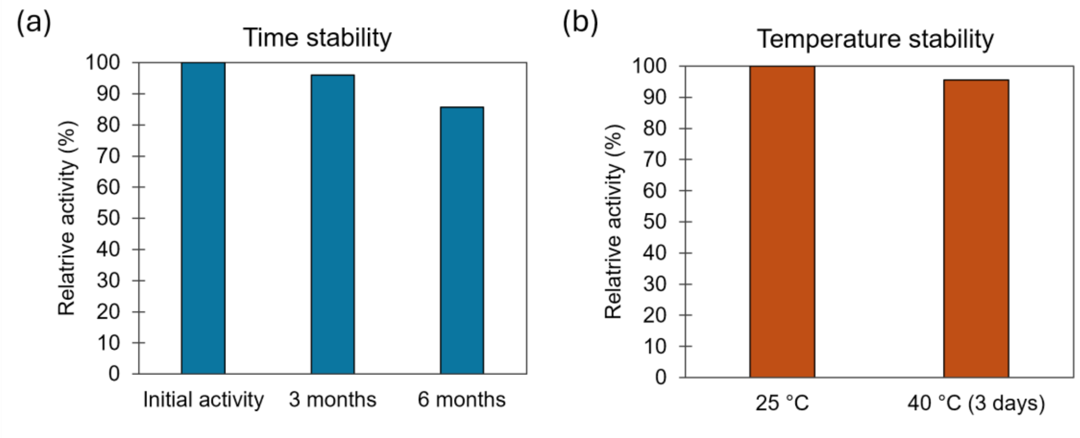

Figure S6. (a) Time and (b) temperature stability of NanoCuAg. The activity was evaluated using the Fenton-like activity in the oxidation of pAP (10 mM) using 3 mg of NanoCuAg in the presence of 0.5% H_2O_2 (v/v).
